# Solitary multicystic lesion lung cancer: two case reports and review of the literature

**DOI:** 10.1186/s12890-021-01729-7

**Published:** 2021-11-14

**Authors:** Xi Tang, Gang Liu, Xianglan Tan, Chengjun Liu, Jin Xiang, Yu Jiang

**Affiliations:** 1 Department of Respiratory and Critical Care Medicine, University-Town Hospital of Chongqing Medical University, Chongqing, 401331 China; 2 Department of Critical Care Medicine, University-Town Hospital of Chongqing Medical University, Chongqing, 401331 China; 3 Department of Thoracic Surgery Department, University-Town Hospital of Chongqing Medical University, Chongqing, 401331 China

**Keywords:** Solitary multicystic lesion lung cancer, Lung cancer associated with cystic airspaces, Case report

## Abstract

**Background:**

Lung cancer associated with cystic airspaces, especially solitary multicystic lesion lung cancer, is a rare disease (a rare imaging performance of non-small cell lung cancer). It is difficult to diagnose owing to the lack of a clear definition; therefore, diagnosis of these neoplastic lesions remains challenging.

**Case presentation:**

We outlined two cases of elderly Chinese men who were admitted to the hospital with a solitary multicystic lesion of the lung and subsequent surgical resection, confirming a diagnosis of adenocarcinoma.

**Conclusions:**

For solitary pulmonary cystic airspaces (especially solitary multicystic lung lesions), it is important to properly recognise their imaging features. Due to the possibility of malignancies, timely surgery is an effective treatment strategy for early diagnosis.

## Background

Lung cancer associated with cystic airspaces (LCCA) is an uncommon manifestation, representing only 1–7% of all lung cancers [1]. Solitary multicystic lesion lung cancer is classified as an adenocarcinoma with the lowest incidence of LCCA [2, 3]. In contrast to the common imaging manifestations of masses or nodules in lung cancer, LCCA is mainly characterised by a cystic area (single or multicystic), with associated consolidation and/or ground-glass opacity [4].

After Womack and Graham first suggested that pulmonary cystic lesions are possibly associated with bronchogenic carcinoma in 1941, Anderson and Pierce reported on carcinoma of the bronchus presenting as thin-walled cysts for the first time in 1954 [2]. Since then, LCCA has been described as a rare disorder owing to the lack of a clear definition for this condition [5]. Coupled with the relatively small number of clinical cases, there is a risk of missed diagnosis or misdiagnosis in the early stage, resulting in a delayed treatment period [6]. Therefore, familiarisation with LCCA and diagnosis and treatment of this special type of lung cancer has become an important goal of our future research.

In this case report, we present two male patients admitted to the hospital with a solitary multicystic lung lesion who had undergone surgical resection, confirming a diagnosis of adenocarcinoma. A review of the conditions focusing on their diagnosis and treatment is also included.

## Case presentation

### Case 1

A 66-year-old man, who had smoked 20 cigarettes per day for 40 years, presented to the pulmonary department with sudden onset haemoptysis for 1 week. He had other concurrent symptoms, including a chronic wet cough for 20 years, with no associated dyspnoea, chest pain, or weight loss. The patient had no history of any other disease. On initial physical examination, he had a blood pressure of 154/105 mmHg, with a pulse rate of 77 beats per minute (bpm). He had normal heart sounds and clear lungs with no dry or wet rales on auscultation.

**Fig. 1:**
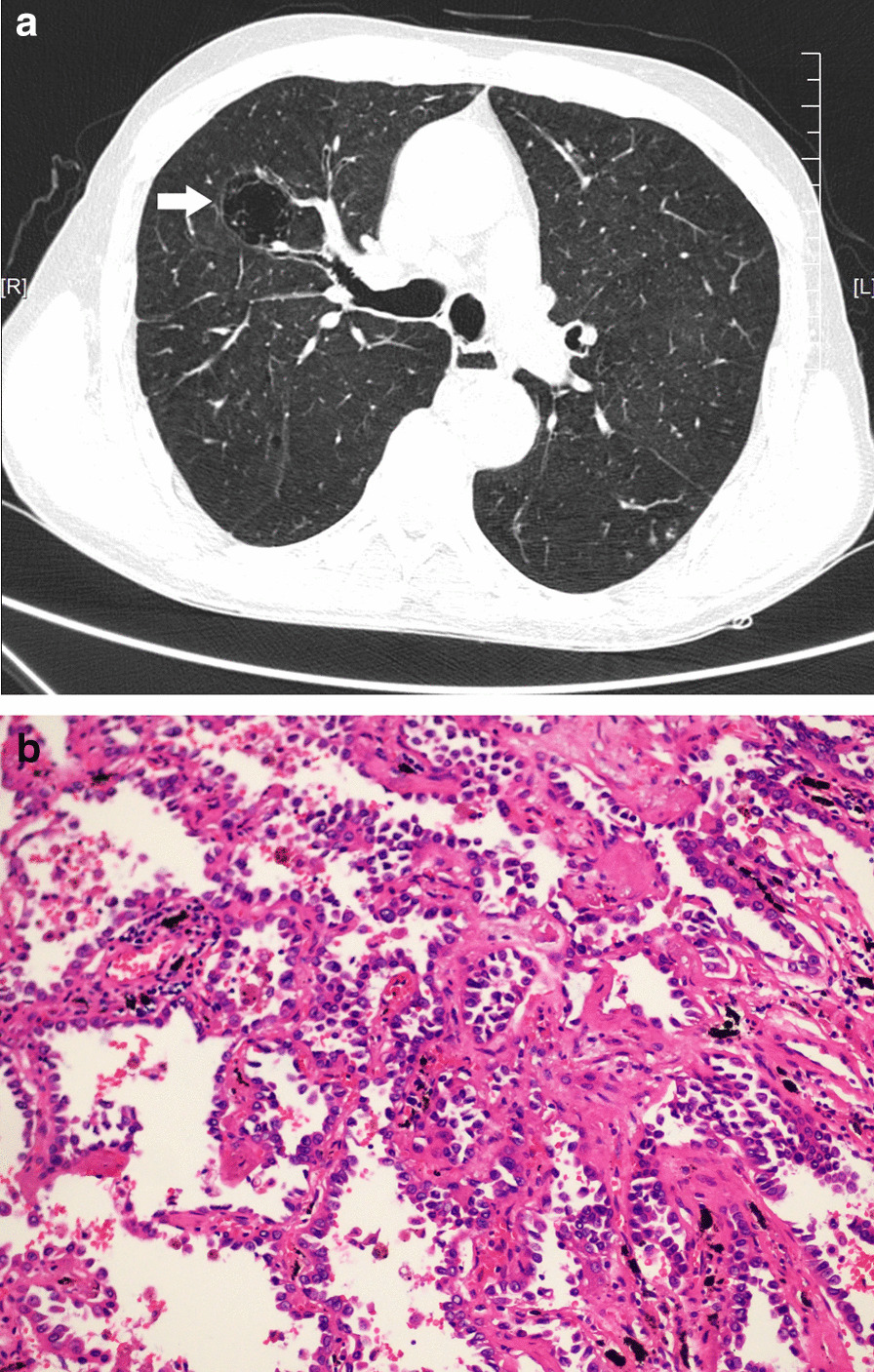
**a** Chest computed tomography demonstrated a solitary multicystic lesion with thin walls in the right upper lobe on September 23, 2019. **b** The histopathology confirmed it to be an adenocarcinoma. (Hematoxylin and eosin, ×200.)

Routine laboratory investigations were normal, including complete blood count, serum urea, and electrolyte levels. Tumour markers (carcinoembryonic antigen [CEA], squamous cell carcinoma antigen [SCC-Ag], and neuron-specific enolase [NSE]) were all within normal limits. Chest computed tomography (CT) demonstrated a solitary multicystic lesion with a thin-wall measuring 24 mm × 12 mm in the right upper lobe (Fig. 1), with non-solid nodules along the cyst wall.

A pulmonary function test revealed a forced expiratory volume in 1 s as the percentage of the predicted value (FEV1%pred) of 61% and a ratio of FEV1/forced vital capacity (FVC) reduced to 58.6% (< 70%), suggesting an obstructive ventilatory disturbance due to smoking; this indicates that the patient has chronic obstructive pulmonary disease (COPD).

Two days later, flexible bronchoscopy revealed only blood clots, and bronchoalveolar lavage in the right upper lobe bronchus found neither malignant cells nor acid-fast bacilli (AFB). Noting that the patient already had haemoptysis and that other diseases commonly causing haemoptysis (i.e., bronchiectasis) were not initially considered, a percutaneous CT-guided biopsy of the solitary multicystic lesion was performed two days later. Since the cystic airspace was completely thin-walled, two small pieces of lung tissue were penetrated. Following tissue biopsy fixation, processing, sectioning and routine histochemical staining, histological examination showed alveolar epithelial hyperplasia (AEH) with an adherent growth pattern without excluding adenocarcinoma in situ, microinvasive adenocarcinoma, or invasive adenocarcinoma due to the lack of tissue.

Due to the small amount of tissue acquired with biopsy under transthoracic puncture, the pathological result did not present an exact diagnosis of LCCA. However, the presence of atypical AEH cells raised speculation regarding early LCCA. Surgery is one of the most reliable therapies for pulmonary adenocarcinomas. We subsequently performed a right upper lobectomy with lymph node dissection using video-assisted thoracoscopic surgery (VATS). Grossly, the sliced resected specimen revealed a tan-whitish cystic tumour, 2.3 cm in length, scattered with small nodules. However, lung cancer was not identified in the frozen section, supporting the diagnosis of a benign mass.

Three days later, a histopathology report documented lepidic-predominant adenocarcinoma with 40% invasive acinar adenocarcinoma. (Fig. 1). The lesion was completely excised. Based on the above findings, the patient was diagnosed with stage IIB pulmonary adenocarcinoma according to the 8th edition of the tumour node metastasis (TNM) staging system for lung cancer. After the operation, the patient was treated with platinum-based adjuvant chemotherapy for four cycles. The postoperative course was uneventful, and there was no recurrence 24 months post-surgery.

### Case 2

**Fig. 2:**
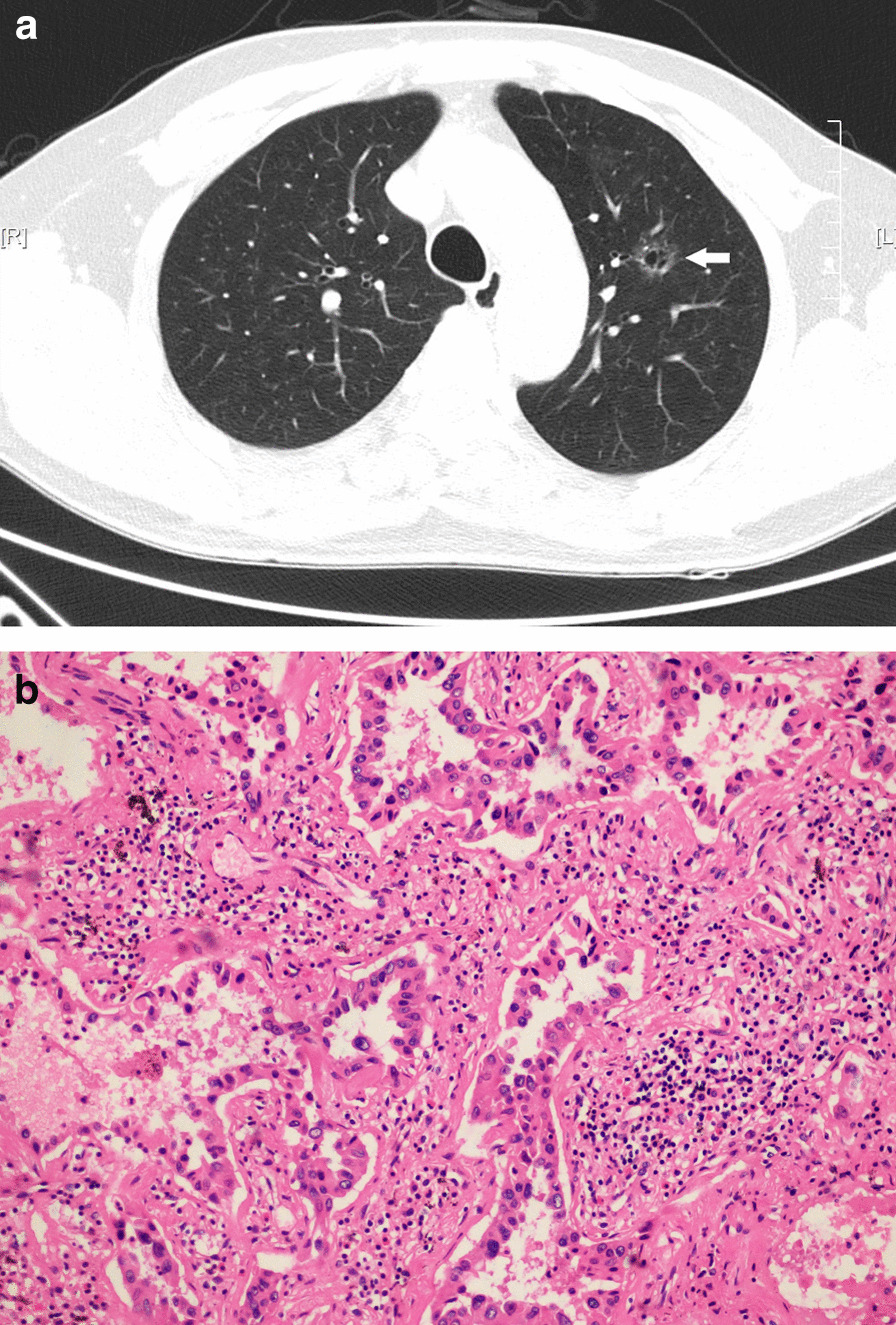
**a** Lung-window of computed tomography revealed a cyst in the left upper lung on August 10, 2020. **b** The histopathology confirmed it to be an adenocarcinoma. (Hematoxylin and eosin, ×200.)

Patient 2 was a 56-year-old man who had no smoking history and presented to the pulmonary department with no symptoms. Radiological examination revealed a cyst measuring 10 mm × 8 mm in the left upper lung (Fig. 2). The doctor in our department included LCCA as a differential diagnosis due to the previous case and suggested that the patient undergo further diagnostic tests for clarification. Laboratory findings showed elevated serum levels of NSE (18.07 ng/mL, normal 0–16.30 ng/mL), CEA, and cytokeratin fragment 21-1 (CYFRA21-1), whereas other routine laboratory investigations and pulmonary function test results were normal. After the failure of percutaneous CT-guided biopsy of the solitary multicystic lesion, a left upper lobectomy was performed using video-assisted thoracoscopic surgery (VATS).

The pathological results showed that invasive adenocarcinoma was found in the cyst region, and almost all components were acinar (Fig. 2). The lesion was completely excised. After the operation, he did not receive any additional chemotherapy and recovered well.

## Discussion and conclusions

Lung cancer associated with cystic airspaces (LCCA) has been reported since the 1940s; however, it is still currently considered as a relatively rare tumour (a rare imaging performance of NSCLC). In 2017, Fintelmann et al. [7] showed that LCCA accounts for approximately 1% of NSCLC, with the majority classified as adenocarcinoma and found in former and current smokers with pulmonary emphysema.

As LCCA has an overall low prevalence, it is easy for radiologists and respiratory doctors to miss it [8]. Patients with LCCA tend to have a worse prognosis than those with non-cystic airspaces lung cancer. Early diagnosis may be challenging, and the condition may already be advanced at the time of detection.

Therefore, CT image features and possible carcinogenic mechanisms may help us understand and diagnose cystic airspace cancers [7, 9]. Currently, only a few retrospective studies have analysed radiological or pathological diagnostic criteria or the management of this condition [10, 11]. In the current radiological system categorising all LCCA, four morphologic types of pericystic cancers have been described by Mascalchi et al. [3, 12]: Type I represents a nodule outside the cystic airspace and abutting the wall. Type II is that of a nodule projecting into the cystic airspace from the wall. Type III is cyst wall thickening, which may not necessarily be circumferential and without an area of focal nodularity. Type IV is a multicystic lesion with focal soft-tissue elements. The two cases, which have been described, belong to this special type of classification system. In addition, Jung et al. [13] showed us the imaging morphological changes in LCCA at different developmental stages, reflecting the natural clinical course and clinicopathological features of LCCA. The true pathogenesis of the cystic airspace is not yet fully understood; however, the different causes have been described [14–18], including (1) central necrosis of tumour; (2) a check-valve mechanism of small airway dilatation with scar tissue; (3) direct destruction of alveolar walls by lung cancer cells; (4) lepidic growth of adenocarcinoma on emphysematous lung parenchyma; (5) cancer arising from clusters of mucinous cells in the walls of this type of congenital pulmonary airway malformation; (6) growth of adenocarcinoma along the wall of a preexisting bulla; (7) and autophagy of cancer cells.

Cystic airspaces are not always present. Most LCCA lesions have new or increased nodular components or thickening of the wall over time. Sheard et al. [5] reported some cases which showed thickening around the periphery of a bulla, and the bulla had progressed to an eccentric or solid nodule. CT scans showed a continued growth of the bulla that was initially misinterpreted as an inflammation, which later became multicystic before evolving into a solid lesion. Mascalchi [12] reported on the comparison of initial and subsequent CTs and revealed that as the solid component increased, the diameter of the cystic airspace decreased or became completely solid in most of his patients. This duration can explain why we misunderstood the initial CT images of the LCCA. In these patients, the risk of death was increased since we were not familiar with the early stages of the tumour, which is the cystic airspace. If we fully understand the CT image development process of the disease, early diagnosis and initiation of treatment in LCCA may alter disease outcomes [19]. The mortality rates of early stage lung cancer resection remain low. Fintelmann et al. [7] reported that the median time between the first observation of a cystic airspace and lung cancer diagnosis was 25.5 months. Thus, cystic airspaces with wall thickening and/or associated nodules of any attenuation warrant regular surveillance. Simultaneously, thoracoscopic surgery is indispensable as a minimally invasive approach for patients with highly suspicious lung cancer.

After a CT scan shows the initial examination of cystic airspaces, appropriate further radiological and invasive operations may be required for the diagnosis of LCCA. Normal clinical inspection such as transbronchial biopsy, percutaneous lung biopsy, and positron emission tomography-computed tomography (PET-CT) are helpful but insufficient for special types such as type IV multicystic lesion, as mentioned in the two cases [20].

Mendoza et al. found that more than 300 cases of LCCA have been described [11] in the form of case reports or small case series; most cases have been diagnosed by surgery in the literature. Farooqi et al. [10] identified 26 cases of abutting lung cysts, and 25 cases were diagnosed by resection. Guo et al. [21] identified 15 cases of “lung cancer presenting as cysts”; all were from surgical resection cases. However, a different conclusion has been drawn by Mascalchi et al. [3, 12], who reported 24 cases of lung cancer associated with cystic airspaces, and the diagnosis of malignancy was based on cytologic examination on CT-guided fine-needle aspiration biopsy (FNAB) (n = 18) or histologic diagnosis of the surgical specimen or core biopsy (n = 6) [5]. In clinical practice, FNAB has been shown to be a safe and effective method for establishing a diagnosis of pulmonary solid and subsolid lesions [22]. However, for LCCA, especially type IV, it often ends in failure due to the small size of the lesion and the difficulty of sampling, as shown in case 2. Therefore, we should recognise the limitations of preoperative biopsy in the diagnosis of these patients.

In contrast, PET-CT has a number of drawbacks in LCCA. Small and cystic air space lesions, multicystic type IV in particular, may reduce the overall density of metabolically active cells; hence, the uptake of fluorine-18-fluorodeoxyglucose (FDG) in a small pericystic lesion can be difficult to measure, and a negative PET result does not reliably exclude malignancy [9, 12, 23, 24].

We report two cases of type IV LCCA that showed multicystic lesions. With increased physician suspicion and improved CT scan and puncture biopsy techniques, the diagnosis rate of LCCA has increased, but it is still often overlooked and misdiagnosed. The majority of cancers associated with cystic airspaces are of the adenocarcinoma type. Awareness of these CT features is important, and the limitations of preoperative biopsy and PET-CT should be recognised. Timely surgery is essential for early diagnosis.

## Data Availability

The datasets used and/or analysed during the current study are available from the corresponding author upon reasonable request.

## Abbreviations

LCCA: Lung cancer associated with cystic airspaces
NSCLC: Non-small cell lung cancer
bpm: Beats per minute
CEA: Carcinoembryonic antigen
SCC-Ag: Squamous cell carcinoma antigen
NSE: Neuron-specific enolase
CT: Computed tomography
FEV1: A forced expiratory volume in 1 s
FVC: Forced vital capacity
COPD: Chronic obstructive pulmonary disease
AFB: Acid-fast bacilli
AEH: Alveolar epithelial hyperplasia
VATS: Video-assisted thoracoscopic surgery
TNM: Tumour node metastasis
CYFRA21-1: Cytokeratin fragment 21-1
PET-CT: Positron emission tomography-computed tomography
FNAB: Fine-needle aspiration biopsy
FDG: Fluorodeoxyglucose
